# Late initiation of combination antiretroviral therapy in Canada: a call for a national public health strategy to improve engagement in HIV care

**DOI:** 10.7448/IAS.18.1.20024

**Published:** 2015-10-05

**Authors:** Angela Cescon, Sophie Patterson, Colin Davey, Erin Ding, Janet M Raboud, Keith Chan, Mona R Loutfy, Curtis Cooper, Ann N Burchell, Alexis K Palmer, Christos Tsoukas, Nima Machouf, Marina B Klein, Sean B Rourke, Anita Rachlis, Robert S Hogg, Julio SG Montaner, the CANOC Collaboration

**Affiliations:** 1 British Columbia Centre for Excellence in HIV/AIDS, Vancouver, Canada; 2 Northern Ontario School of Medicine, Sudbury, Canada; 3 Faculty of Health Sciences, Simon Fraser University, Burnaby, Canada; 4 Faculty of Science, St. Francis Xavier University, Antigonish, Canada; 5 Toronto General Research Institute, University Health Network, Toronto, Canada; 6 University of Toronto, Toronto, Canada; 7 Women's College Research Institute, Toronto, Canada; 8 Department of Medicine, University of Ottawa, Ottawa, Canada; 9 Ontario HIV Treatment Network, Toronto, Canada; 10 Faculty of Medicine, McGill University, Montreal, Canada; 11 Clinique Médicale l'Actuel, Montreal, Canada; 12 Sunnybrook Health Sciences Centre, Toronto, Canada; 13 Division of AIDS, Department of Medicine, University of British Columbia, Vancouver, Canada

**Keywords:** HIV, AIDS, antiretroviral therapy, late initiation, public health, HIV care, Canada

## Abstract

**Introduction:**

Combination antiretroviral therapy (ART) significantly decreases morbidity, mortality and HIV transmission. We aimed to characterize the timing of ART initiation based on CD4 cell count from 2000 to 2012 and identify factors associated with late initiation of treatment.

**Methods:**

Participants from the Canadian Observational Cohort (CANOC), a multi-site cohort of HIV-positive adults initiating ART naively after 1 January 2000, in three Canadian provinces (British Columbia, Ontario and Québec) were included. Late initiation was defined as a CD4 count <200 cells/mm^3^ or an AIDS-defining illness before ART initiation (baseline). Temporal trends were assessed using the Cochran–Armitage test, and independent correlates of late initiation were identified using logistic regression.

**Results:**

In total, 8942 participants (18% female) of median age 40 years (Q1–Q3 33–47) were included. The median baseline CD4 count increased from 190 cells/mm^3^ (Q1–Q3 80–320) in 2000 to 360 cells/mm^3^ (Q1–Q3 220–490) in 2012 (*p*<0.001). Overall, 4274 participants (48%) initiated ART with a CD4 count <200 cells/mm^3^ or AIDS-defining illness. Late initiation was more common among women, non-MSM, older individuals, participants from Ontario and BC (vs. Québec), persons with injection drug use (IDU) history and individuals starting ART in earlier calendar years. In sub-analysis exploring recent (2008 to 2012) predictors using an updated CD4 criterion (<350 cells/mm^3^), IDU and residence in BC (vs. Québec) were no longer significant correlates of late initiation.

**Conclusions:**

This analysis documents increasing baseline CD4 counts over time among Canadians initiating ART. However, CD4 counts at ART initiation remain below contemporary treatment guidelines, highlighting the need for strategies to improve earlier engagement in HIV care.

## Introduction

Published literature provides extensive evidence that delayed initiation of combination antiretroviral therapy (ART) in HIV infection increases the risk of poor treatment outcomes, morbidity and mortality [1–4]. Despite substantial evidence supporting the benefits of early initiation of ART, HIV continues to be diagnosed and treated later than contemporary guidelines recommend [2, 5–7].

Alongside the significant personal health benefits, ART regimens have been used to prevent vertical HIV transmission to infants for decades, by suppressing plasma HIV-RNA concentration to undetectable levels [8–10]. More recently, ART has also been shown to elicit the benefit of preventing HIV transmission via sexual and parenteral routes [11–15]. At the community and population levels, expanded access to ART and reductions in aggregate HIV-RNA measures have been shown to correlate with decreasing new HIV diagnoses [16–18].

As a key component of the HIV “cascade of care” [19], a better understanding of temporal trends in the timing of ART initiation across Canada is required, as this has yet to be quantified for this region. Identifying factors that support or undermine timely initiation of treatment can help guide the development of improved HIV testing and healthcare engagement strategies. To these ends, we characterized the timing of ART initiation and identified correlates of late treatment initiation across three Canadian provinces from 2000 to 2012.

## Methods

### Design and setting

This study was conducted within the Canadian Observational Cohort (CANOC) collaboration, a multi-site study of HIV-positive individuals initiating ART after 1 January 2000 [20]. Currently, eight cohorts contribute data to CANOC, representing the country's three most populous provinces (Ontario, British Columbia (BC) and Québec). CANOC is the largest collaborative HIV cohort in Canada, aiming to gain an improved understanding of trends in ART use and HIV treatment outcomes. Nearly half of the estimated 20,500 people on HIV treatment in the represented provinces are captured in this cohort [21].

For inclusion in CANOC, participants must be at least 18 years old, have documented HIV infection, reside in Canada, have initiated an ART regimen comprising at least three individual antiretroviral agents naively (i.e., no prior antiretroviral experience) on or after 1 January 2000, and have recorded CD4+ T-lymphocyte (CD4) cell count and HIV-RNA plasma viral load testing results within six months of ART initiation.

Data extraction is performed locally at the participating sites and is then pooled and analyzed at the BC Centre for Excellence in HIV/AIDS in Vancouver. All participating cohorts have received ethical approval from their institutional boards to contribute data to this collaboration. Further details on the collaborating cohorts and general CANOC structure have been published previously [20]. The last date of follow-up in the cohort for the current analysis was 31 December 2012.

### Outcomes and statistical methods

Outcomes of interest included [1] timing of ART initiation, determined by the CD4 cell count of participants at first initiation of ART, and [2] late ART initiation, defined as having a pre-ART (baseline) CD4 cell count <200 cells/mm^3^ or an AIDS-defining illness. In a sub-analysis examining more recent (2008 to 2012) trends in ART initiation, the CD4 cell count cut-off for late initiation was increased to <350 cells/mm^3^ in order to reflect contemporary HIV treatment guidelines [22–24].

Baseline demographic and clinical characteristics of participants were summarized using medians and interquartile ranges (Q1–Q3) for continuous variables and frequencies and proportions for categorical variables. Categorical characteristics were compared between late and non-late initiators using the Pearson χ^2^ or Fisher's exact test, and continuous variables using the Wilcoxon rank-sum test. Variables of interest included age, gender, province, ethnicity, HIV risk factors, hepatitis C virus (HCV) co-infection, baseline plasma viral load and year of ART initiation.

Temporal trends in the timing of ART initiation were assessed using the Cochran–Armitage test and factors independently associated with late initiation were determined using logistic regression. Variables with statistical significance of *p*<0.05 in bivariate analyses and those considered important based on clinical hypothesis were candidates for inclusion in the final multivariable models. In the instance of covariates measuring similar phenomena (such as injection drug use (IDU) history and HCV co-infection), the variable representing each construct with the higher effect size and most statistical significance was selected. Statistical analyses were performed using SAS software, version 9.3.

## Results

### Study population

In total 8942 individuals were included in this study, with 1634 (18%) female participants. The median age at pre-ART baseline was 40 years (Q1–Q3 33–47), baseline CD4 cell count 220 cells/mm^3^ (Q1–Q3 120–327) and baseline plasma viral load 4.9 log_10_ copies/mL (Q1–Q3 4.4–5.0). Almost half (49%, *n*=4360) of participants resided in BC. Of 8356 individuals with HCV testing results, 2192 (26%) were seropositive. The majority of participants initiated ART on NNRTI-based (46%) or boosted PI-based (44%) regimens, most frequently regimens containing efavirenz (36%) or atazanavir (23%).

### Timing of ART initiation

**Figure 1:**
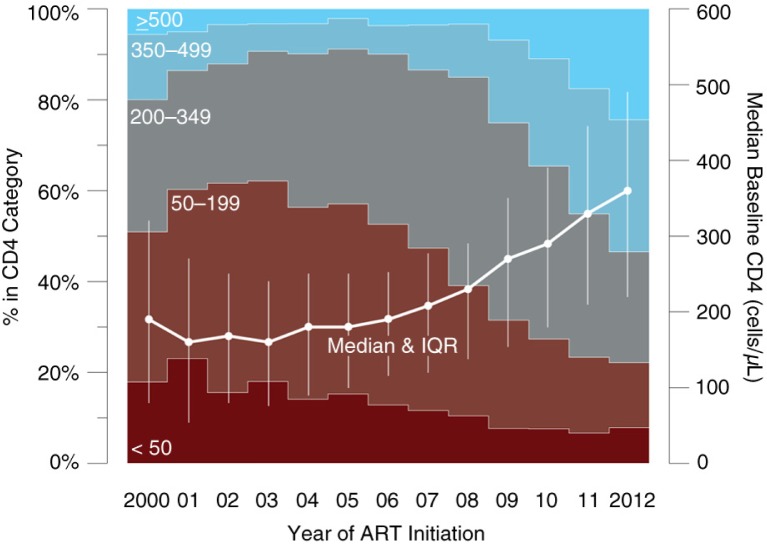
Distribution and median of CD4 cell counts at ART initiation in CANOC, 2000–2012 (*n*=8942). ART, combination antiretroviral therapy; IQR, interquartile range (Q1–Q3).

**Figure 2:**
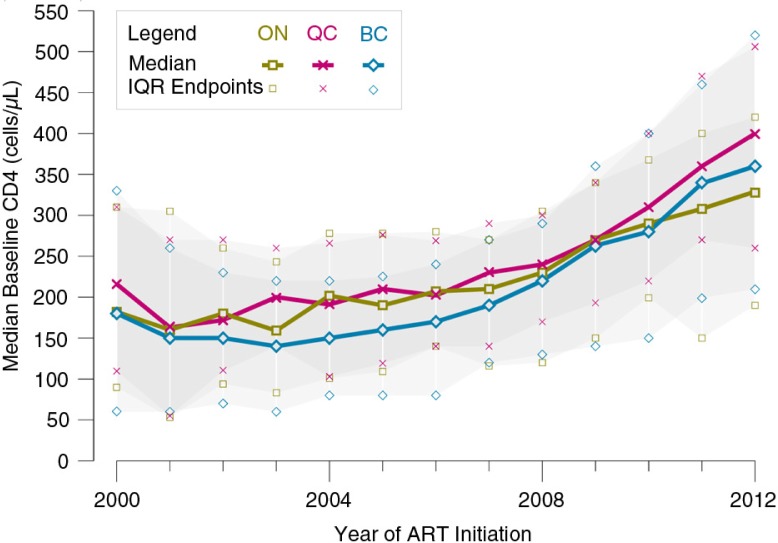
Median baseline CD4 cell counts at ART initiation by province, 2000–2012 (*n*=8942). ART, combination antiretroviral therapy; IQR, interquartile range (Q1–Q3); ON, Ontario; QC, Québec; BC, British Columbia.

The median baseline CD4 cell count increased from 190 cells/mm^3^ (Q1–Q3 80–320) in 2000 to 360 cells/mm^3^ (Q1–Q3 220–490) in 2012 (89% increase, *p*<0.001). Of the 664 participants initiating ART in the most recent study year (2012), 22% (*n*=147) started with a CD4 cell count <200 cells/mm^3^, and 162 (24%) with a CD4 cell count between 200 and 349 cells/mm^3^ (Figure 1). The highest baseline CD4 cell counts at ART initiation in 2012 were observed among participants from Québec (median [Q1–Q3] 400 [260–506] cells/mm^3^), followed by BC (360 [210–520] cells/mm^3^) and Ontario (329 [190–420] cells/mm^3^) (Figure 2).

### Late ART initiation

Overall, 48% of participants (*n*=4274) initiated ART with a CD4 cell count <200 cells/mm^3^ or a baseline AIDS-defining illness. In a sub-analysis focussing exclusively on participants initiating ART in the time period 2008 to 2012 (*n*=4048), 68% of participants (*n*=2,751) initiated ART with a CD4 cell count <350 cells/mm^3^ or with a baseline AIDS-defining illness. Among the 664 participants initiating ART in 2012, the proportion with a baseline CD4 cell count <350 cells/mm^3^ or an AIDS-defining illness was 48% (*n*=316).

In bivariate analysis, late ART initiators differed significantly from non-late initiators in terms of age, gender, province, ethnicity, HIV risk factors, HCV serostatus, year of ART commencement and baseline HIV-RNA viral load (Table 1).

**Table 1:** Demographic and clinical characteristics of CANOC participants at pre-ART baseline, late vs. non-late ART initiators (2000–2012) (*n*=8942)

Variable	Total	Non-late *n*=4668	Late^a^ *n*=4274	*p*
Gender				
Female	1634	807 (17)	827 (19)	0.012
Male	7308	3861 (83)	3447 (81)	
Age (years)				
18–29	1259	810 (17)	449 (11)	<0.001
30–39	3048	1584 (34)	1464 (34)	
40–49	3087	1515 (32)	1572 (37)	
≥50	1548	759 (16)	789 (18)	
Province				
British Columbia	4360	2181 (47)	2179 (51)	<0.001
Ontario	2705	1351 (29)	1354 (32)	
Québec	1877	1136 (24)	741 (17)	
Ethnicity				
Caucasian	2,467	1165 (25)	1302 (30)	<0.001
Black	788	327 (7)	461 (11)	
Aboriginal ancestry	435	168 (4)	267 (6)	
Other	677	318 (7)	359 (8)	
Unknown	4575	2690 (58)	1885 (44)	
IDU history				
No	5284	2872 (62)	2412 (56)	<0.001
Yes	2004	838 (18)	1166 (27)	
Unknown	1654	958 (21)	696 (16)	
MSM^b^				
No	1862	755 (20)	1107 (32)	<0.001
Yes	3373	1909 (49)	1464 (42)	
Unknown	2073	1197 (31)	876 (25)	
HCV co-infection				
No	6164	3396 (73)	2768 (65)	<0.001
Yes	2192	969 (21)	1223 (29)	
Unknown	586	303 (6)	283 (7)	
Initial third ARV class^c^				
NNRTI	4122	2378 (51)	1744 (41)	<0.001
Single PI	505	261 (6)	244 (6)	
Boosted PI	3933	1793 (38)	2140 (50)	
Other	382	236 (5)	146 (3)	
Initial third ARV^c^				
Nevirapine	831	427 (9)	404 (9)	<0.001
Efavirenz	3177	1856 (40)	1321 (31)	
Lopinavir	1451	542 (12)	909 (21)	
Atazanavir	2024	1072 (23)	952 (22)	
Other	1459	771 (17)	688 (16)	
Year ART initiated	8942	2008 (2005–2010)	2006 (2003–2008)	<0.001
CD4 count (cells/mm^3^)	8942	310 (250–409)	110 (46–170)	<0.001
Viral load (log_10_ copies/mL)	8942	4.66 (4.19–5.00)	5.00 (4.66–5.00)	<0.001

Results are *n* (%) or median (Q1–Q3). ART, combination antiretroviral therapy; IDU, injection drug use; HCV, hepatitis C virus; MSM, men who have sex with men; ARV, antiretroviral; NNRTI, non-nucleoside reverse transcriptase inhibitor; PI, protease inhibitor.

a Late initiation defined as baseline CD4 cell count <200 cells/mL or baseline AIDS-defining illness

b among *n*=7308 men

c alongside two NRTIs.

In adjusted multivariable analysis spanning the entire 12-year study period, late initiation was more common among women and non-MSM (vs. MSM), older individuals, participants from Ontario and BC (vs. Québec), persons with IDU history and individuals starting ART in earlier calendar years (Table 2). In sub-analysis exploring recent predictors of late initiation from 2008 onwards using the updated CD4 cell count criterion of <350 cells/mm^3^, similar trends were observed for all covariates with the exceptions of province and IDU history. In this sub-analysis, late initiation was more common among women and non-MSM (vs. MSM), older individuals, participants from Ontario (vs. Québec), and individuals starting ART in earlier calendar years; IDU history and residence in BC (vs. Québec) were no longer significant predictors of late initiation (Table 2).

**Table 2:** Factors associated with late ART initiation in CANOC, 2000–2012 and 2008–2012 (*n*=8,942)

Variable	2000–2012^a^				2008–2012^b^			
	
	Unadjusted OR (95% CI)	*p*	Adjusted OR (95% CI)	*p*	Unadjusted OR (95% CI)	*p*	Adjusted OR (95% CI)	*p*
Age (years)								
18–29	1.00 (−)	<0.001	1.00 (−)	<0.001	1.00 (−)	<0.001	1.00 (−)	<0.001
30–39	1.67 (1.46, 1.91)		1.58 (1.37, 1.82)		1.47 (1.21, 1.79)		1.47 (1.19, 1.80)	
40–49	1.87 (1.64, 2.14)		1.85 (1.60, 2.13)		1.59 (1.31, 1.93)		1.59 (1.29, 1.96)	
≥50	1.88 (1.61, 2.18)		1.95 (1.66, 2.29)		2.08 (1.66, 2.61)		2.15 (1.68, 2.73)	
Province								
Québec	1.00 (−)	<0.001	1.00 (−)	<0.001	1.00 (−)	<0.001	1.00 (−)	<0.001
British Columbia	1.53 (1.37, 1.71)		1.49 (1.31, 1.70)		1.08 (0.91, 1.27)		0.94 (0.76, 1.17)	
Ontario	1.54 (1.36, 1.73)		1.68 (1.47, 1.91)		1.49 (1.23, 1.80)		1.50 (1.21, 1.86)	
Gender+MSM								
Male, MSM	1.00 (−)	<0.001	1.00 (−)	<0.001	1.00 (−)	<0.001	1.00 (−)	<0.001
Female	1.34 (1.19, 1.50)		1.27 (1.11, 1.45)		1.60 (1.30, 1.96)		1.75 (1.39, 2.21)	
Male, non-MSM	1.91 (1.70, 2.14)		1.59 (1.39, 1.83)		1.81 (1.48, 2.21)		1.74 (1.36, 2.22)	
Male, unknown	0.95 (0.85, 1.07)		1.02 (0.86, 1.20)		0.91 (0.77, 1.06)		1.30 (1.00, 1.68)	
IDU history								
No	1.00 (−)	<0.001	1.00 (−)	0.007	1.00 (−)	<0.001	1.00 (−)	0.018
Yes	1.66 (1.49, 1.84)		1.15 (1.00, 1.31)		1.43 (1.19, 1.73)		1.08 (0.84, 1.37)	
Unknown	0.87 (0.77, 0.97)		0.83 (0.71, 0.97)		0.73 (0.62, 0.86)		0.71 (0.56, 0.91)	
Year ART initiated^c^	0.86 (0.85, 0.87)	<0.001	0.86 (0.85, 0.87)	<0.001	0.62 (0.59, 0.66)	<0.001	0.63 (0.60, 0.67)	<0.001

ART, combination antiretroviral therapy; OR, odds ratio; CI, confidence interval; IDU, injection drug use; MSM, men who have sex with men.

a Late initiation defined as baseline CD4 cell count <200 cells/mL or baseline AIDS-defining illness

b late initiation defined as baseline CD4 cell count <350 cells/mL or baseline AIDS-defining illness

c odds ratio per incremental year of calendar time.

## Discussion

We report a significant increase in CD4 cell counts at primary ART initiation among participants of a large Canadian HIV cohort study over a 12-year study period (2000 to 2012). However, CD4 cell counts at ART initiation remain below contemporary treatment guidelines [24, 25], with nearly half (48%) of participants initiating ART in 2012 starting with a baseline CD4 cell count <350 cells/mm^3^ or an AIDS-defining illness. Furthermore, our analysis identified important correlates of late ART initiation. These findings contribute to a better understanding of the timing of ART commencement across Canada and support the expansion of improved healthcare engagement strategies.

As expected, over the study period we documented a shift towards earlier initiation of ART, demonstrated by the 89% increase in the median pre-ART CD4 cell count from 190 cells/mm^3^ in 2000 to 360 cells/mm^3^ in 2012. This trend mirrors temporal changes in HIV treatment guidelines over the period of observation. Since the introduction of combination HIV therapies the optimal time to initiate ART in asymptomatic patients has been a debated issue [26], historically with concerns around the potential for ART toxicities and resistance [27]. However, international HIV and health governing bodies including the World Health Organization and the International AIDS Society (IAS) now offer widespread and consistent support for initiating treatment earlier in the course of infection [24, 28]. Specifically, while international HIV treatment guidelines have evolved over the past decade, the IAS and the United States Department of Health and Human Services (DHHS) now recommend that all adults with HIV infection be offered ART immediately following diagnosis, irrespective of CD4 cell count. Furthermore, results of the 2015 multi-continental randomized START trial offer unequivocal empirical evidence that immediate ART initiation, regardless of CD4 cell count, is superior to deferral of therapy [29]. To note, there are presently no Canada-wide therapeutic guidelines. Provincial HIV/AIDS management guidelines exist in BC [30] and Québec [31], whereas guidelines of the DHHS are followed in Ontario.

Important correlates of late ART initiation identified in this study include residence in Ontario or BC (vs. Québec), female gender and non-MSM history, older age, IDU history and earlier year of ART initiation. When more recent (2008 to 2012) trends were assessed using an updated CD4 cell count criterion, findings remained unchanged with the exceptions of the differences between participants with and without IDU history, and from BC and Québec, which were no longer statistically significant.

Differences by province should be interpreted cautiously as a selection bias exists whereby data from BC include the entire sample of CANOC-eligible individuals province-wide due to the population-based HIV treatment registry. Comparatively, data from Ontario and Québec are obtained from a selection of specialized HIV clinics, the majority being in urban centres. As such, these data may not represent all CANOC-eligible persons on ART in these provinces; however, the majority of individuals living with HIV in Ontario and Québec reside in these areas [20]. More importantly, significant improvements were documented in all jurisdictions over the study period.

Differences by gender and sexual orientation observed in this analysis are consistent with a number of reports in the contemporary literature suggesting that women may be more likely to be diagnosed at clinically advanced stages of HIV infection and delay ART initiation [32–35], and that late presentation is common among heterosexual men [35, 36]. Poorer access to HIV care services and suboptimal treatment outcomes among women have been previously reported, in both Canadian and comparable international settings [34, 37–40]. This observation is important to consider in the context of the changing epidemiology of Canada's HIV epidemic. Echoing global trends, there has been a gradual incidence escalation among Canadian women over the last 15 years, with women accounting for 24% of new positive tests among adults in 2011 (double the proportion of 12% observed from 1985 to 1998) [41].

In CANOC, a significantly higher proportion of women than men report a history of IDU [38], which reflects the epidemiology of the Canadian HIV epidemic, particularly in BC. Indeed, IDU may pose additional psychosocial and structural barriers to accessing HIV testing and engaging with HIV care, including mental health and addiction issues, food insecurity, housing challenges and other comorbid conditions [42]. Our findings allude to the importance of prioritizing low-threshold services that aim to alleviate barriers to accessing HIV testing and ART for individuals who use injection drugs [42, 43].

Previous studies in other global settings have similarly found older age to be associated with later ART initiation [34]. Within our analytic sample, we hypothesize that later ART initiation among older adults may relate to decreased HIV testing behaviours, possibly due to a lower perception of risk. It is also possible that older adults in our cohort may encounter barriers to accessing care that are not captured in this analysis.

While unable to quantify the contribution of late HIV diagnosis to the high prevalence of late ART initiation observed in this study, it is prudent to consider this an important contributing factor. A recent report from BC estimated this contribution to be almost 70% [44]. As in other settings, undiagnosed HIV infections remain a significant concern in Canada, with the Public Health Agency of Canada (PHAC) estimating that 25% of HIV-positive Canadians are unaware of their status [41]. Diagnosing patients at the earliest possible stage of HIV infection is critical to optimize the personal and public health benefits of ART. Late ART initiators often present with complex clinical circumstances, making treatment more challenging. Furthermore, direct healthcare costs in the year following HIV diagnosis have been documented in excess of 200% higher for patients with CD4 cell counts <200 cells/mm^3^ [45], highlighting the substantial economic impacts of late presentation for HIV care.

### Limitations

When contextualizing these results important limitations should be considered. CANOC includes data from only three provinces and results may not be generalizable to all HIV-positive individuals in Canada. Also, as mentioned, entry of participants into the study differs by province. Data from BC include the entire sample of CANOC-eligible individuals province-wide, while data from Ontario and Québec come from a selection of clinics that are mainly HIV-specific. Finally, we did not examine differences in the timing of ART initiation by ethnicity in multivariable analysis due to a high proportion of missing data. Regional analyses in some Canadian jurisdictions have reported significant variation in the timing of ART initiation by ethnicity and Aboriginal ancestry [46, 47]. Future studies exploring such differences are thus warranted in order to identify any such inequities at the pan-provincial level.

## Conclusions

In Canada, the offering of HIV testing during routine clinical encounters remains largely based on perceived risks (“risk-based” testing). Continued use of these testing practices may hinder diagnosis opportunities (particularly in light of the demographic shifts in Canada's epidemic) and contribute to ongoing stigmatization related to HIV testing [48]. The authors note that incorporating HIV testing into routine medical care is embraced in PHAC's 2013 HIV Screening and Testing Guide [49], however to our knowledge testing mandates of this kind have to date only been implemented in BC. Province-wide initiatives in BC aim to normalize routine HIV testing in effort to achieve more timely diagnosis and subsequent linkage to care [50]. Specific efforts have included outreach to family physicians clarifying the new testing paradigms, routine HIV testing in hospitals and in primary care clinics, and media campaigns. Results of the current study provide impetus for the implementation of improved HIV testing paradigms across all regions of Canada (tailored to each region's unique epidemiology and healthcare context), in effort to decrease the burden of undiagnosed HIV, reduce onward HIV transmission, and encourage earlier initiation of treatment and engagement in the HIV care cascade to allow the optimal benefits of ART to be realized.

In conclusion, our results demonstrate that CD4 counts at ART initiation remain below contemporary treatment guidelines. These findings contribute to a better understanding of the timing of ART commencement across Canada, and should help to inform the implementation of improved healthcare engagement strategies. We advocate for the development of Canadian consensus guidelines based on the UNAIDS 90-90-90 framework (by 2020, 90% of all people living with HIV will know their status, 90% of all people with diagnosed HIV will receive ART, and 90% of people receiving ART will have viral suppression) [51] as a useful next step to provide uniform, evidence-based clinical directives for HIV management across Canada.
